# Assessment of physical activity patterns in patients with rheumatoid arthritis using the UK Biobank

**DOI:** 10.1371/journal.pone.0319908

**Published:** 2025-03-26

**Authors:** Valentin Hamy, Andrew Creagh, Luis Garcia-Gancedo

**Affiliations:** 1 GSK, London, United Kingdom; 2 University of Oxford, Oxford, United Kingdom; 3 GSK, Stevenage, Hertfordshire, United Kingdom; Saga University, JAPAN

## Abstract

Measures of physical activity patterns that may characterize rheumatoid arthritis status were investigated, using actigraphy data from a large, prospective database study (UK Biobank). Population characterization identified 1080 individuals with rheumatoid arthritis who participated in accelerometer-measured physical activity data collection and met the eligibility criteria; these individuals were subsequently matched with 2160 non-rheumatoid arthritis controls. Raw actigraphy data were pre-processed to interpretable acceleration magnitude and general signal-based features were used to derive activity labels from a human activity recognition model. Qualitative assessment of average activity profiles indicated small differences between groups for activity in the first 5 hours of the day, engagement in moderate-to-vigorous activity, and evening sleep patterns. Of 145 metrics capturing different aspects of physical activity, 57 showed an ability to differentiate between participants with rheumatoid arthritis and non-rheumatoid arthritis controls, most notably activities related to moderate-to-vigorous activity, sleep and the ability to perform sustained activity, which remained different when adjusting for baseline imbalances. Objective measures derived from wrist-worn accelerometer data may be used to assess and quantify the impact of rheumatoid arthritis on daily activity and may reflect rheumatoid arthritis symptoms. This work represents an initial step towards the characterization of such impact. Importantly, this study offers a glimpse of the potential use of large-scale datasets to support the analysis of smaller clinical study datasets.

## Introduction

Rheumatoid arthritis (RA) is a chronic inflammatory disease associated with pain, fatigue, and progressive articular damage leading to impaired mobility and ability to perform daily tasks [1–4]. Globally, RA affects 20 million people with an age-standardized prevalence rate of 246.6 per 100,000 and age-standardized incidence rate of 14.9 per 100,000 individuals, making it a major global health issue with an important unmet need in terms of treatment and monitoring [2].

Many symptoms experienced by patients with RA, i.e., pain, fatigue, stiffness, and ability to perform activities of daily living, are difficult to quantify reliably using existing measures. Assessment of these symptoms relies on patient-reported outcomes (PROs) and in-clinic tests, which are known to be limited by aspects such as subjectivity a lack of validity and concerns with their clinical relevance [5–7]. In clinical trials, these limitations can compromise assessment of the potential for new treatments to benefit aspects of daily life that are most important to patients, prescribers, payers, and regulators. Actigraphy devices provide an opportunity to monitor and quantify the impact of these symptoms on daily life activities by recording human activity and mobility resulting from physical activity, sedentary behavior, and sleep [8]. In a clinical trial in RA, a bespoke ensemble of decision tree-based algorithms were used to accurately estimate specific periods of activities (e.g., walking) from real-world free-living actigraphy data [9], demonstrating the potential of actigraphy sensors to be used as a tool to identify activities of daily living of patients with RA. Another study comparing RA patients with control participants matched on BMI, sex and geographical location, using data from hip-worn accelerometers showed that patients with RA were significantly more sedentary during the day due to morning stiffness and fatigue [10].

Prior studies showed that actigraphy can be used to accurately distinguish activity types as well as to characterize activities of daily living of patients with RA [9–16]. While over a dozen studies have shown that actigraphy measures are potentially useful to assess the impact of RA on physical activity [10,12,15–23], this research was conducted using small datasets. To better understand the generalizability of the physical activity metrics and to quantify the impact of symptoms on patients, we studied activity differences between patients with RA and non-RA controls using data from the UK Biobank.

The UK Biobank is a large prospective study that has collected phenotypic and genotypic data from 500,000 participants aged 40 to 69 years when recruited between 2006 and 2010 at 22 assessment centers [24]. As well as comprising a comprehensive range of clinical assessments data, including biological samples, functional measurements and imaging, the dataset contains questionnaire data on lifestyle and socio-demographics, and is linked to electronic health records [25–27]. Between February 2013 and December 2015, participants from the UK Biobank were asked to wear an accelerometer (Axivity AX3) on their dominant wrist for 7 days continuously to record physical activity [28]. Using machine learning methods, sleep behaviors and physical activity were classified with 87% accuracy in UK Biobank participants [29]. More recently, measures of physical activity derived from this accelerometer dataset were used to predict mortality [30] as well as investigate the incidence of Parkinson’s disease [31].

The objective of our work was to identify measures of physical activity that may reflect the impact of RA symptoms on patterns of daily activity in a large-scale population.

## Materials and methods

### Ethics approval

Data access and use for the work described in this manuscript, including for use of research, is managed by the UK Biobank. All data has been accessed through UK Biobank Application #20361 in accordance with the UK Biobank Ethics and Governance framework. General ethical approval for the UK Biobank, including informed consent procedures, was initially granted by the North West Multi-Centre Research Ethics Committee in 2011 and renewed in 2021 (Ref 21/NW/0157). All participants provided written informed consent. Methods reported in this manuscript were performed on anonymized data, in accordance with relevant guidelines and regulations covered by the aforementioned ethics approval committee. UK Biobank data including accelerometer, demographics, and clinical characteristics released by 14.08.2020 was used in this study.

### Study design

Socio-demographic, lifestyle and health-related information for the participants were collected from the UK Biobank database along with information on actigraphy data validity [28]. In this study (conducted under UK Biobank application #20361), we investigated 145 sensor-based measurements capturing daily activity patterns. All processing stages described in the following sections were performed on raw accelerometer time series.

### Study population

Previous work by Siebert et al. conducted in 2016 [27] showed that the prevalence of RA by self-report in the UK Biobank was 1.13% (5657 participants), with a median duration of 10 years (interquartile range 4–20). Approximately half of participants with RA (2849, 50.4%) were taking medication, most of whom (2708, 47.9%) took one or more synthetic disease-modifying antirheumatic drug.

In this study, participants with RA were selected as individuals from the UK Biobank sub-population with recorded actigraphy data who had a primary International Classification of Diseases 10th Revision code of RA: either seropositive RA (M05) or other RA (M06) from primary/secondary care sources or self-reported. A previously defined criterion for actigraphy data validity requiring a minimum of 72 hours of device wear time, was applied [28].

Non-RA controls were matched (2:1) to participants with RA by age, sex, body mass index (BMI), and area deprivation index (ADI), using a nearest-neighbor matching strategy without replacement [32,33]. Participants’ age (calculated from birth year to actigraphy data collection start date) was categorized from 45 to 80 years in 5-year increments, BMI was categorized as underweight (<18.5 kg/m^2^), healthy weight (18.5–25), overweight (25–30), obese (30–40), or severely obese (≥40), and ADI values were categorized into quintiles. The ratio of other potential covariates, such as smoking, alcohol consumption or chronotype, were investigated post-matching.

### Activity levels and bouts

Raw actigraphy sensor data were pre-processed to interpretable acceleration magnitude (based on milligravities [m*g*]). Acceleration levels in 40 m*g* increments (e.g., 0–40 m*g*, 40–80 m*g* up to 400 m*g*) were investigated to obtain greater granularity in the differences in terms of sedentary behaviors which are known to be more prevalent among patients with RA [10] and moderate activity which is significantly reduced for patients with RA [34].

Physical activity bouts were defined as time intervals of sustained activity for more than a minimum threshold duration and intensity. The three types of bouts studied were sedentary (<40 m*g*), active (40– < 100 m*g*) and moderate-to-vigorous physical activity (MVPA; ≥ 100 m*g*) with activity intensity thresholds based on previous work [35–37]. Active and MVPA bouts were computed using a multi-resolution approach similar to that introduced in [38]. Duration thresholds were set to 10 minutes for active bouts and 3 minutes for MVPA bouts. The former was chosen to align with the global recommendation on physical activity by the World Health Organization (WHO) [39] while the latter was heuristically selected to be long enough to ensure a transition from anaerobic to aerobic activity, but also short enough to occur as part of normal daily activities. Sedentary bouts were defined as the time complementary to the active bouts.

### Human activity recognition modeling

Participants’ actigraphy data were pre-processed per standard protocol [29] including data conversion of raw sensor data to interpretable acceleration magnitude. General signal-based features were generated from the acceleration magnitude on the epoch level every 30 seconds and subsequently used to derive activity labels from a random forest human activity recognition (HAR) model [29,40].

The pre-processed acceleration magnitude data were segmented into categories of intensity of activity such as periods of sleep, sedentary, light intensity activity, and MVPA [40].

Consolidated periods of night-time lying down were computed [41,42] on a per night basis whereby the detected sleep periods output by the HAR model were used as input to an iterative correlation procedure. Identification of sustained walking periods or ambulatory bouts were based on walking as detected using an alternative version of the HAR model [29]. Ambulatory bouts were heuristically grouped by durations of 2–10 minutes, 10–30 minutes, and longer than 30 minutes, and allowed for a single break of predefined maximum duration lasting up to 30 seconds (for 2–10 minutes duration) and 1 minute (for 10–30 minutes and longer than 30 minutes duration).

### Metrics of physical activity

The metrics gauged were grouped into four domains: total volume of activity (, morning activity, night-time activity, and fragmentation of activity. A detailed listing of indices of physical activity grouped by ‘domain’ of activity is available in S1 Table. Metrics in the “total volume of activity” domain aim to capture the amount of daytime activity and include summary statistics at different timescales (e.g. daily or weekly) for acceleration magnitude, time and percent of time spent at different activity levels, acceleration magnitude during bouts, time and percent of time spent in bouts at different activity levels, time and percent time spent walking as well as the number of ambulatory bouts of each type.

Metrics in the “morning activity” domain aim to capture information reflecting morning stiffness and include summary statistics of acceleration magnitude across different time durations after getting up (e.g. 1, 2 or 4 hours). The time of getting up was estimated as the end of the consolidated periods of night-time lying down described in the previous section.

Metrics in the “night-time activity” domain aim to capture sleep disturbance and restlessness including sleep efficiency (percentage of time detected as sleep within a given night-time lying down window), circadian-rhythm-related metrics of timing (sleep midpoint, timing of the least-active 5 hours (L5) and the most-active 10 hours (M10)), duration and variability of nocturnal and diurnal sleep episodes [43] along with summary statistics of the number of movement episodes (periods within the night-time lying down window not labeled as sleep by the HAR model) per night and rest fragmentation (percentage of time detected as non-sleep within a given night-time lying down window, divided by the number of movement episodes).

Finally, metrics in the “activity fragmentation” domain aim to measure the ability to perform activity in a sustained way including average hazard (of switching from sedentary to active and vice versa or from non-MVPA to MVPA and vice versa) [44], between-state transition probability [44] and summary statistics of the duration of consecutive time spent in each state.

### Statistical analysis

For each metric the result values were log-transformed to reduce skewness of the distribution in each group and mean relative differences and 95% confidence intervals (CI) were computed. A metric was considered to have a suspected ability to differentiate between participants with RA and non-RA controls if the mean relative difference was > 5% and the 95% CI excluded 1.

Analysis of variance (ANOVA) was performed to assess the statistical differences between RA and non-RA groups and the effects across sources of RA diagnosis (primary/secondary care sources or self-report), diagnosis types (seropositive or other), and disease onset. Disease onset is classified as early or younger onset versus late or elderly onset, depending on whether a patient’s age is lower or greater than (or equal to) 60 years when symptoms appear [45]. In this study, age at the time of the first reported diagnosis was used as a proxy to estimate disease onset. Analysis of covariance (ANCOVA) was used to assess statistical differences between RA and non-RA groups while adjusting for potential covariates such as smoking, alcohol consumption, chronotype (the tendency for a person to be naturally more active in the morning or in the evening, as self-reported by the participant), occupation and the season when actigraphy data were collected. P-values were exploratory and not adjusted for multiplicity.

A principal component analysis (PCA), using data from all participants with RA and non-RA controls, was performed on shortlisted metrics with a suspected ability to differentiate between RA and non-RA groups to better understand the interrelationship in the manifestation of physical activity. The metrics were centered and standardized to have a mean of 0 and a standard deviation (SD) of 1 before carrying out the PCA to prevent results from being driven by the different scales of the metrics.

## Results

### Participant disposition

Of 106,053 participants in the UK Biobank who agreed to wear an accelerometer, 1603 (1.51%) had RA and satisfied the inclusion criteria, 1082 of whom were considered suitable for analysis. Reasons for records being excluded were duplicate RA diagnoses (n = 118) from which only the most tangible outcome was kept (e.g., primary care over self-report, M05 over M06), diagnosis after actigraphy data recording period (n = 249), and history of conditions that might influence physical activity such as heart failure (n = 52), respiratory failure (n = 9), stroke (n = 35), thyroiditis (n = 2), and Sjögren syndrome (n = 74).

Of the 1082 participants with RA, 1080 were successfully matched by age, sex, BMI, and ADI to non-RA controls (n = 2160) from a pool of 89,939 eligible participants, as defined by no RA diagnosis in the health record, no history of heart failure, respiratory failure, stroke, thyroiditis, or Sjögren syndrome, and excluding participants tested for rheumatoid factor with a concentration above the normal range (>20 IU/mL). A flow diagram summarizing participants numbers is available in supplementary material (S1 Fig)

### Demographics and clinical characteristics

RA participant demographics and clinical characteristics are reported in **Tables 1 and 2**. The mean (SD) age of participants was 64.33 (7.11) years and most were aged ≥ 60 years (74.6%), female (67.0%), and had early disease onset (74.0%). After matching, imbalances between participants with RA and non-RA controls included fewer smokers, less alcohol consumption, more evening chronotypes, greater autumn and less summer/winter actigraphy recording, and lower working rates among participants with RA (S2 Fig), each of which was included as a covariate in the ANCOVA for comparison of activity types between RA and non-RA groups.

**Table 1:** Demographics of UK Biobank participants with RA who provided actigraphy data and met eligibility criteria.

	No. of participants (%)	
	Seropositive RA	Other RA^*^
Total	94	988
**Age, years**		
45– < 50	3 (3.2)	34 (3.4)
50– < 55	4 (4.3)	86 (8.7)
55– < 60	10 (10.6)	138 (14.0)
60– < 65	24 (25.5)	183 (18.5)
65– < 70	29 (30.9)	281 (28.4)
70– < 75	20 (21.3)	231 (23.4)
75–80	4 (4.3)	35 (3.5)
**Female**	73 (77.7)	652 (66.0)
**Male**	21 (22.3)	336 (34.0)
**Body mass index, kg/m** ^**2**^		
<18.5	3 (3.2)	4 (0.4)
18.5– < 25	27 (28.7)	305 (30.9)
25– < 30	40 (42.6)	406 (41.1)
30– < 40	21 (22.3)	246 (24.9)
≥40	3 (3.2)	23 (2.3)
**Area deprivation index** ^†^		
Least deprived quintile	21 (22.3)	195 (19.7)
2nd quintile	14 (14.9)	202 (20.5)
3rd quintile	24 (25.5)	192 (19.4)
4th quintile	20 (21.3)	196 (19.8)
Most deprived quintile	15 (16)	201 (20.3)
**Source**		
Primary care	8 (8.5)	48 (4.9)
Primary care (plus other)	5 (5.3)	35 (3.5)
Hospital admissions	77 (81.9)	212 (21.5)
Hospital admissions (plus other)	4 (4.3)	13 (1.3)
Self-report	0 (0)	389 (39.4)
Self-report (plus other)	0 (0)	291 (29.5)

* Primary/secondary care sources or self-reported.

† The area deprivation index values for the RA population (mean: -1.48, SD: 2.88) were discretized into equal-sized buckets based on sample quintiles which were then used in the matching.

RA, rheumatoid arthritis; SD, standard deviation.

**Table 2:** Disease characteristics of UK Biobank participants with RA who provided actigraphy data and met eligibility criteria.

30 (3.0)	No. participants (%)	
	Seropositive RA	Other RA^*^
Total	94	988
**Medication type**		
CSDMARDS	48 (51.1)	294 (29.8)
CSDMARDS (plus other)	6 (6.4)	33 (3.3)
Steroids	3 (3.2)	27 (2.7)
BDMARDS (plus other)	4 (4.3)	12 (1.2)
BDMARDS	0 (0)	(0.3)
Other/Unknown	33 (35.1)	619 (62.7)
**Disease onset** ^†^		
Early	53 (56.4)	748 (75.7)
Late	41 (43.6)	240 (24.3)
**Smoking status**		
Ever	36 (38.3)	471 (47.7)
Never	57 (60.6)	511 (51.7)
Unknown	1 (1.1)	6 (0.6)
**Alcohol consumption**		
Daily	16 (17.0)	168 (17.0)
3–4 times per week	15 (16.0)	197 (19.9)
1–2 times per week	25 (26.6)	243 (24.6)
1–3 times per month	5 (5.3)	133 (13.5)
Rarely	19 (20.2)	143 (14.5)
Never	14 (14.9)	103 (10.4)
**Chronotype**		
Morning person	50 (53.2)	538 (54.5)
Evening person	28 (29.8)	323 (32.7)
Unknown	16 (17.0)	127 (12.8)
**Comorbidity**		
Depression^‡^	13 (13.8)	133 (13.5)
Knee osteoarthritis	13 (13.8)	108 (10.9)
Benign prostatic hyperplasia	2 (2.1)	46 (4.7)
Sleep apnea	4 (4.3)	34 (3.4)
Gout	3 (3.2)	30 (3.0)
Diabetes	2 (2.1)	9 (0.9)

* Primary/secondary care sources or self-reported. ^†^First RA symptoms appeared before (early) or after age 60 (late). ^‡^Recurring or single episode.

BDMARDS, biologic disease-modifying antirheumatic drugs; CSDMARDs, conventional systemic disease-modifying antirheumatic drugs; RA, rheumatoid arthritis.

### Population average activity profiles

**Fig 1:**
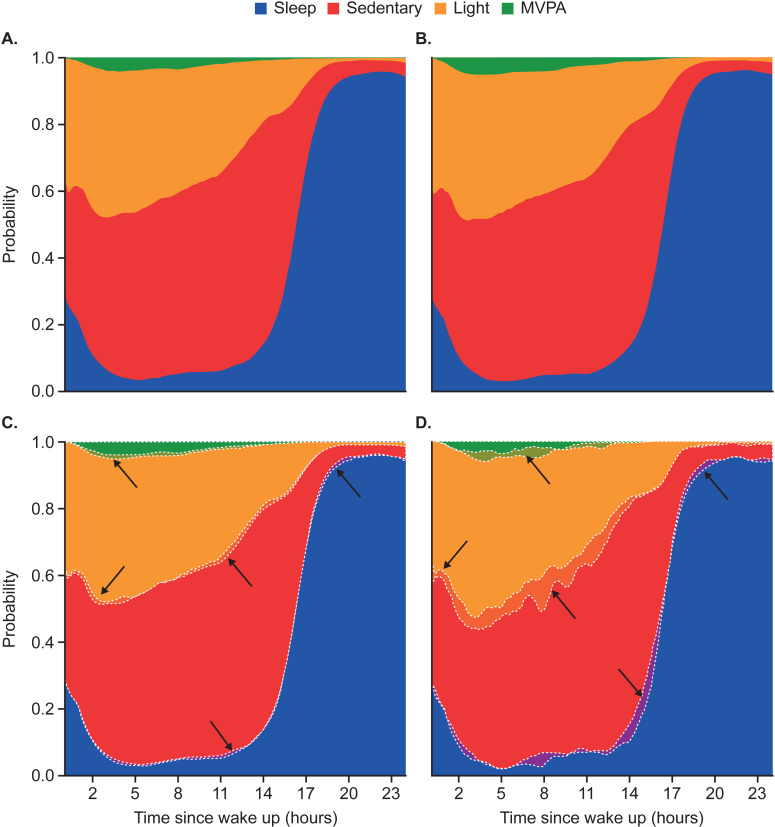
Population average activity profiles for (A) participants with RA, (B) non-RA controls, (C) difference between the two profiles (highlighted by dotted lines and arrows) and (D) differences between profiles for RA and non-RA subgroups who were not working and had morning chronotype; the RA subgroup excludes self-report diagnosis and early disease onset. MVPA, moderate-to-vigorous physical activity; RA, rheumatoid arthritis.

The probability of a physical activity type (sleep, sedentary, light activity and MVPA being performed with respect to time since estimated wake-up, averaged for both RA and matched non-RA groups were presented as stack plots (**Fig 1**).

Qualitative assessment of the two groups’ average activity profiles indicated a high degree of similarity, largely dominated by circadian rhythmicity. However, when the average activity profiles for each cohort were overlaid, participants with RA had a lower probability than non-RA controls for activity in the first 5 hours of the day and engagement in MVPA (**Fig 1C**). Activity of participants with RA appeared less likely to be labeled as sleep than that of non-RA controls

17–23 hours after waking. Consistent observations with more pronounced differences were apparent in the average activity profiles for participants who did not work and had morning chronotype (**Fig 1D**).

### Metrics of physical activity

**Fig 2:**
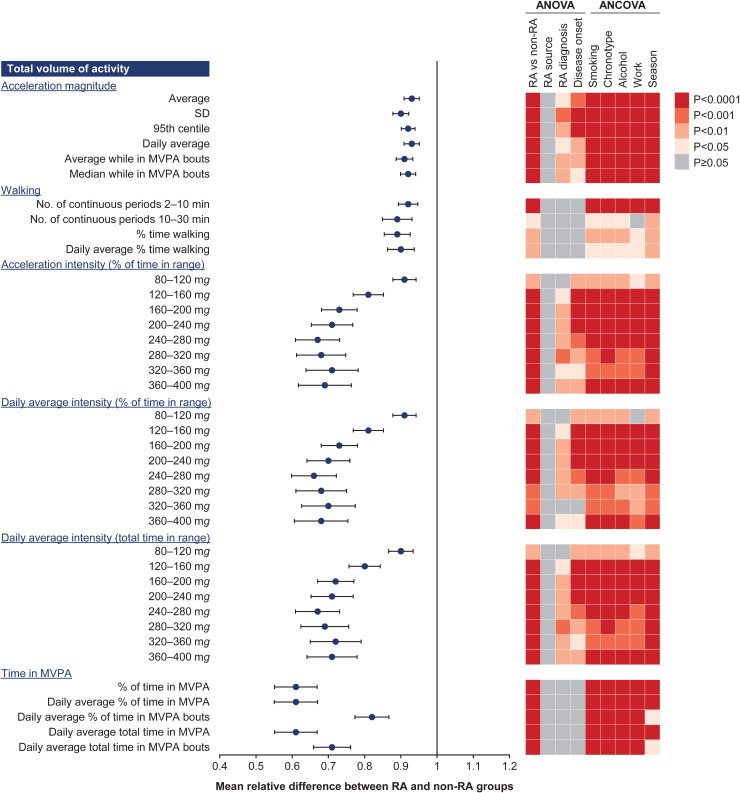
Mean relative difference between groups expressed as the ratio of participants with RA to non-RA controls for relevant significant metrics * in the total volume of activity domain. * Non-significant metrics are not included. ANCOVA, analysis of covariance; ANOVA, analysis of variance; MVPA, moderate-to-vigorous physical activity; RA, rheumatoid arthritis; SD, standard deviation.

**Fig 3:**
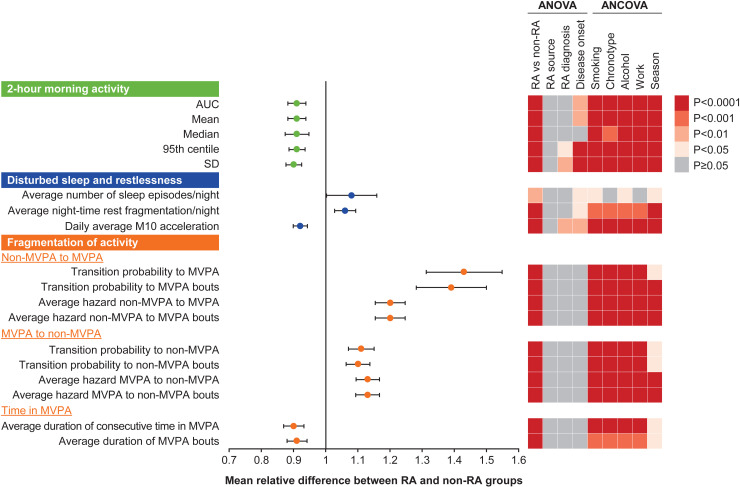
Mean relative difference between groups expressed as the ratio of participants with RA to non-RA controls for relevant significant metrics in the morning activity (morning stiffness), night-time activity (disturbed sleep and restlessness) and fragmentation of activity domains. ANCOVA, analysis of covariance; ANOVA, analysis of variance; AUC, area under the curve; MVPA, moderate-to-vigorous physical activity; M10, midpoint time of the most-active 10 hours; RA, rheumatoid arthritis; SD, standard deviation.

In total, 145 metrics were derived from each RA and non-RA participant’s actigraphy time series, covering several aspects of physical activity across four domains: total volume of activity, morning activity (morning stiffness), night-time activity (disturbed sleep and restlessness), and fragmentation of activity (S1 Table). Summaries of the metrics across all domains that were identified as promising through the assessment of groupwise mean relative difference are presented in **Figs 2** and **3**. Among 77 computed metrics in the total volume of activity domain, which captured information related to the overall daily physical activity of individuals at daily and weekly levels, 42 showed a potential ability to differentiate between participants with RA and non-RA controls (mean relative difference of at least 5% in either direction with a 95% CI excluding 1) and were kept for further analysis. The largest differences were for activities related to MVPA (≥100 m*g*) and were most pronounced for categories of intensity in the range 160 m*g* to 400 m*g* (**Fig 2**). Metrics related to the number of walking bouts captured small differences between the two groups.

Metrics in the morning activity domain captures information related to morning stiffness, a hallmark symptom in patients with RA. Within the morning activity domain, all but three of the 30 metrics showed potential differentiation between groups. Because of redundancy across different time windows (from 15 minutes to 4 hours), only the 2-hour window, which captured the largest differences, was considered. Within the night-time activity (disturbed sleep and restlessness) domain, three of 15 metrics showed a potential differentiation between groups, two of which were related to nocturnal sleep interruptions, and one was related to circadian rhythmicity (average level of activity during the most active 10-hour period of the day). Within the fragmentation of activity domain (metrics related to the ability to perform sustained activity), 10 of 25 metrics showed potential differentiation between groups, all of which were linked to MVPA, with the greatest difference apparent for transitions from non-MVPA to MVPA (**Fig 3**).

For the 60 metrics with potential for differentiation, a 1-way ANOVA showed all values differed statistically between RA and non-RA groups except for “Daily average magnitude while in MVPA bouts”, “Daily average magnitude while in MVPA”, and “Number of continuous walking periods >30 minutes” (**Figs 2**, **3, and** S3 Fig). No statistical difference was found across sources of RA diagnosis, while differences were found for 30 metrics across diagnosis types and for 36 metrics across disease onset types (**Figs 2 and 3**). Comparison of metrics between RA and non-RA groups remained statistically different when adjusting for covariates, except for “Average number of sleep episodes” when adjusting for chronotypes and work status and “Number of continuous walking periods 10–30 minutes” when adjusting for work status.

### Principal component analysis

To explore the interrelationship of physical activity metrics, PCA was performed using data from all participants, excluding 11 metrics due to missing data. A moderate to strong correlation was observed between metrics within the same domain with the strongest correlation for variations in definition of the same measurement. Metrics from different domains were weakly correlated (S4 Fig).

**Fig 4:**
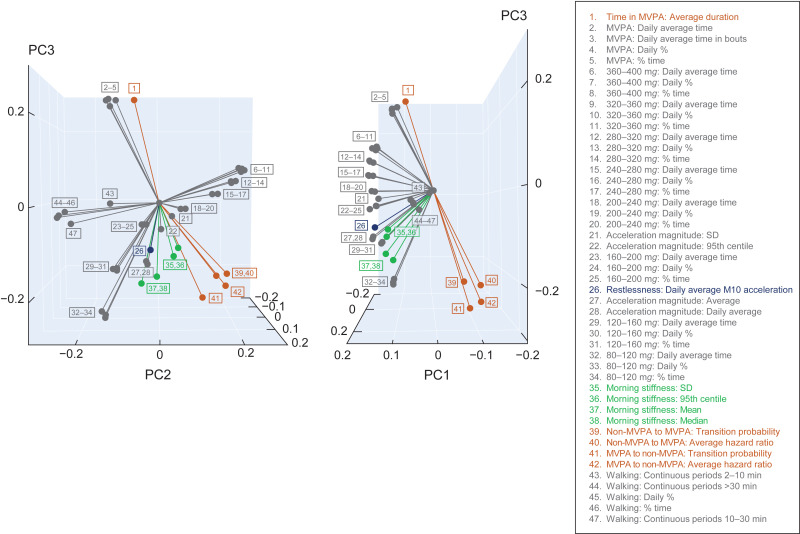
Biplot of the PCA showing loadings of variables (colored by domain) on the first three PCs: (A) and (B) presents different orientations of the 3D space defined by the PCs. MVPA, moderate-to-vigorous physical activity; M10, midpoint time of the most-active 10 hours; PC, principal component; PCA, principal component analysis; SD, standard deviation.

A heuristic cumulative variance threshold of 70% was considered for selecting the principal components (PCs) resulting from the PCA. The first three PCs, which comprised 69.8% of the total variance (S5 Fig), were chosen as a trade-off between meeting the cumulative variance criterion and maintaining interpretability through graphical representation. The contributions of 47 metrics to the three PCs are presented as a biplot in **Fig 4**. The projection of each metric on each axis reflects the loading of the metric on that PC. Short segments have small contributions to the PCs, whereas long segments have large contributions to at least one of the first three PCs. The angle between segments reflects the correlation between the corresponding metrics.

The first octant (all PCs > 0) includes all metrics of activity in the 240–400 m*g* ran*g*es at 40 m*g* increments (metrics 6–17 in Fig 4). Individuals with PCA scores falling in this region will generally present higher-moderate average activity levels, with PC1 decreasing and PC2/3 increasing as the average intensity of activity increases.

The second octant (PC1 and 2 > 0, PC3 < 0) includes a mixture of metrics related to SD and 95th centile of activity either in the morning or daily (metrics 21–22, 35–36), along with metrics of activity in the 200–240 m*g* ran*g*e (metrics 18–20 in Fig 4). Individuals with PCA scores falling in this region would present a high overall variability of activity across mornings and more generally across days, with lower-moderate activity level on average.

## Discussion

This analysis of the physical activity monitoring (actigraphy) data from the UK Biobank showed how a series of objective measures can be derived from wrist-worn accelerometer data to assess and quantify the impact of RA on daily activity, at scale.

Population-level physical activity patterns were characterized via human activity recognition modeling [29,40], allowing for inference of activity types, which showed small yet clear differences between RA and non-RA populations at certain times of day suggesting RA symptoms impact patients’ ability to perform activities of daily living. Participants with RA appeared to be more sedentary in the early hours of the day than non-RA controls (which may be attributable to morning stiffness), less likely to engage in MVPA throughout the day (which may be due to pain, stiffness and fatigue), and sleep less in the night (which may indicate increased restlessness). Average activity profiles may have been driven by circadian rhythmicity with influences from heterogeneous lifestyles and disease sources for the RA group. More pronounced differences in physical activity patterns were observed when participants with RA with a clinically confirmed diagnosis and late disease onset (i.e., more likely to involve large joints) were considered among a more homogeneous group of participants who were not working and had a morning chronotype. Periods of activities such as walking are impacted by RA-induced joint pain and may have variable intensity; however, independent consideration of average walking probability profiles indicated that the difference between RA and non-RA individuals was consistent across the day and unlikely to account for differences in physical activity patterns at specific times of day. However, metrics characterizing periods of continuous walking and percentage of time spent walking did highlight differences.

Metrics measured in domains which assessed total volume of activity, morning activity (morning stiffness), night-time activity (disturbed sleep/ restlessness) and fragmentation of activity offered valuable insights on the impact of the RA on daily activities; this is in alignment with findings in other disease areas including knee/hip arthroplasty [46], Parkinson’s Disease [47], amyotrophic lateral sclerosis [48] or chronic obstructive pulmonary disease (COPD) [49]. Participants with RA generally presented with a lower volume of daily activity and morning activity as well as more fragmented daily activity and disturbed night-time rest which suggests the need to assess these measures using actigraphy devices; lack of sufficient physical activity [50] and disturbed sleep have been associated with poor health-related quality of life in patients with RA [51]. Further, these results align with clinical expectations of RA effects on physical activity [52] and indicate potential for such metrics (considered independently or grouped into composite measures) to capture the effect of a treatment on activity patterns over time. This is consistent with observations for other chronic diseases in participants of the UK Biobank, in which accelerometer-measured physical activity revealed that participants with 67/147 individual disease diagnoses were linked to less moderate physical activity per week than participants without chronic diseases [53]. The UK Biobank is one of largest, most mature datasets currently available. It includes a large sample of actigraphy data which is actively used by researchers from around the world for public health-related investigations and also for the development of novel data analysis tools [54] or as a benchmark for further analysis. Despite its relative age (collected in 2013-2015), the tri-axial accelerometer technology used is still an accepted standard measurement type. While the actigraphy data used in this work is cross-sectional, the UK Biobank continuously expands and updates its datasets. Future data collection initiatives could include follow-up physical activity monitoring, thus offering opportunities for longitudinal extension of the existing actigraphy dataset.

Measures of fragmentation of MVPA seemed to capture the largest differences and, although the probability of transitioning from non-MVPA to MVPA seemed higher for participants with RA, duration of MVPA was shorter in the RA group, suggesting that this may be related to difficulty engaging in sustained activity, which may be useful to incorporate in measures to distinguish patients with RA from individuals without RA.

Findings from the PCA analysis suggest that the first three principal components can be associated with, in order of decreasing importance: overall time spent in physical activity regardless of intensity (50% variance), fragmentation of physical activity (11.1% variance), and intensity of physical activity (8.7% variance). Future work could use a similar representation on a longitudinal dataset to evaluate changes in physical activity patterns over time and to potentially characterize sensitivity to symptoms progression or treatment effects.

The ability of the wrist-worn accelerometer to assess and objectively quantify the impact of RA on daily activity shows promise, detecting differences in sedentary and MVPA by both human activity recognition modeling, allowing for inference of activity types. The reproducibility of these findings was investigated in a digital pilot study of a selected group of patients with RA (WeaRAble-PRO study 212295), using actigraphy data for the metrics that showed promise in characterizing the impact of RA with other information, including PRO measures [55]. The results of that separate study largely aligned with those described in this paper, for instance, activity fragmentation measures reflecting the probability of transitioning from non-MVPA to MVPA, as well as the duration of sustained MVPA, appearing as strong contributors to the differentiation between RA and non-RA participants. Likewise, the population average activity profiles for the RA and non-RA group in the WeaRAble-PRO study [55] presented the same (more pronounced) difference as those highlighted in Fig 1C**-**D.

The UK Biobank permitted a robust investigation of novel digital measures thanks to its large size. However, it presented several important limitations including: 1) post hoc identification of RA among study participants is limited by incomplete clinical information on disease history and severity, thereby potentially resulting in a more heterogeneous population than smaller study; and 2) the study does not capture RA-specific PROs, and hence we were unable to assess individual symptoms such as fatigue, pain, and stiffness. Despite these limitations clear differences in physical activity patterns were identified between participants with RA and non-RA controls, and were not impacted by source of RA diagnosis (primary, secondary care, or self-report), type of diagnosis (seropositive or other RA), or disease onset. However, further evaluation of the considered measures of physical activity patterns in terms of construct validity and clinical meaningfulness could not be completed.

Using the large-scale actigraphy dataset from the UK Biobank we identified several physical activity measures that are indicative of RA and have utility for both hypothesis generation and broad comparison of activity patterns between disease and non-disease groups. Identifying measures that can differentiate between disease and control groups represents a useful step in the investigation of novel digital biomarkers. Further similar exploration of this dataset, combined with a more targeted RA-specific small-scale dataset from a clinical trial in a targeted disease population [23,55], has potential to support development of novel digital endpoints for use to assess disease progression. Future work will investigate how the combination of these multi-scale datasets may be analysed to provide valuable information for the assessment of longitudinal treatment effect on RA and thus may support the development of novel medicine.

This work constitutes an initial step towards this goal and the dual exploration of large-scale/small-scale datasets as an iterative process whereby hypotheses generated in one dataset could be tested or validated in the other. We believe this conceptual approach will pave the way towards demonstrating reliability, validity and sensitivity in development of digital biomarkers.

## Supporting information

S1 Fig Exclusion flow diagram for analysis of UK Biobank physical activity monitoring participants with RA. RA, rheumatoid arthritis. (PDF)

S2 Fig Bar plots representing the proportion of participants with RA and non-RA controls categorized by (A) smoking status (B) alcohol consumption frequency (C) chronotype (D) season when actigraphy data were collected, and (E) aggregate type of occupation. RA, rheumatoid arthritis. (PDF)

S3 Fig Mean relative difference between groups expressed as the ratio of participants with RA to non-RA controls for non-significant metrics in total volume of activity domain. ANCOVA, analysis of covariance; ANOVA, analysis of variance; MVPA, moderate-to-vigorous physical activity; RA, rheumatoid arthritis. (PDF)

S4 Fig Heatmap of the correlation matrix between the 60 metrics of physical activity identified as potentially differentiating between participants with RA and non-RA controls. The magnitude of correlation is represented by both the color and the size of each square. accel., acceleration; AUC, area under the curve; avg., average; cont., continuous; m*g*, milligravities; MVPA, moderate-to-vigorous physical activity; M10, midpoint time of the most-active 10 hours; prob., probability; RA, rheumatoid arthritis. (PDF)

S5 Fig Scree plot of the first 15 PCs resulting from the PCA of the computed metrics of physical activity. PC, principal component; PCA, principal component analysis. (PDF)

S1 Table Indices of physical activity grouped by ‘domain’ of activity pre-processed data type. mg, milligravities; MVPA, moderate-to-vigorous physical activity; SD, standard deviation. (DOCX)
