# Supplementary figures and images for: Assessment of physical activity patterns in patients with rheumatoid arthritis using the UK Biobank
Source: PLoS One. 2025 Mar 26;20(3):e0319908. doi: 10.1371/journal.pone.0319908 (PMC11940758; doi:10.1371/journal.pone.0319908)

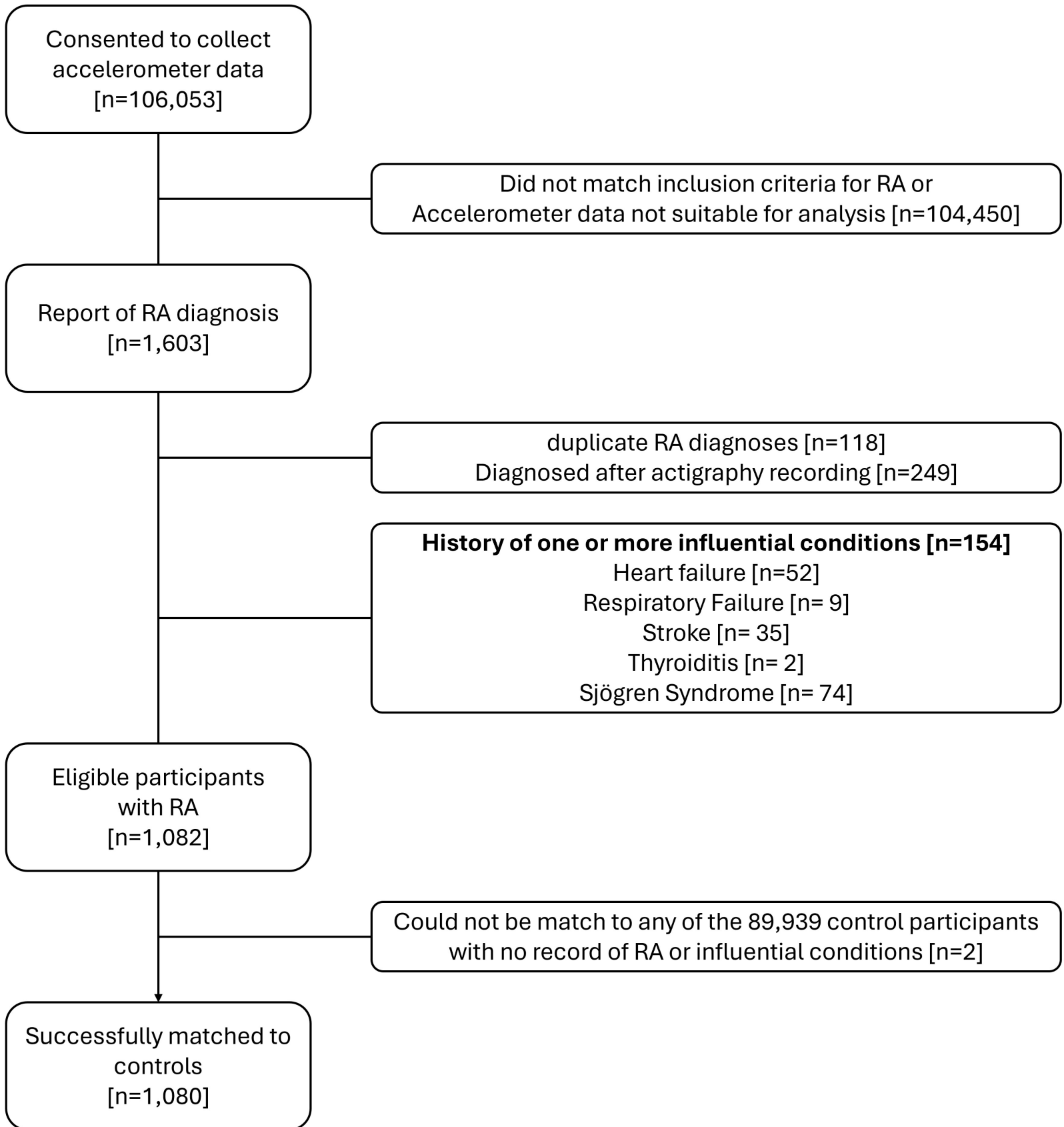

Supplement: S1 Fig — (PDF) [file pone.0319908.s001.pdf]

**A. Smoking Status**

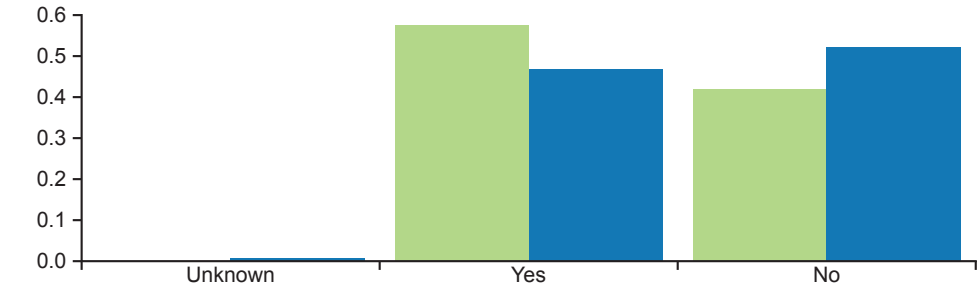

**B. Alcohol Consumption**

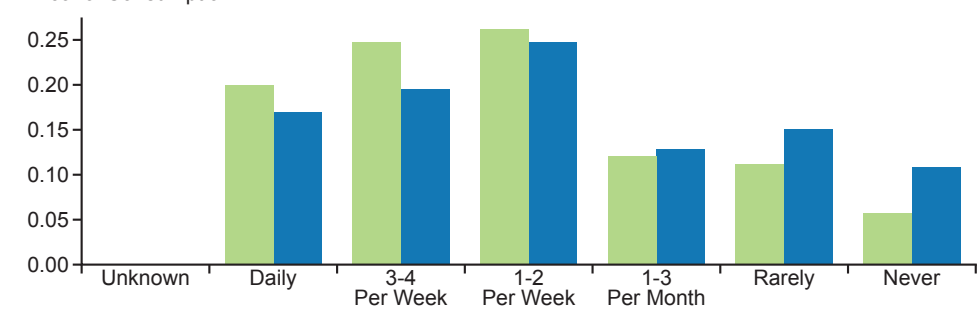

**C. Chronotype**

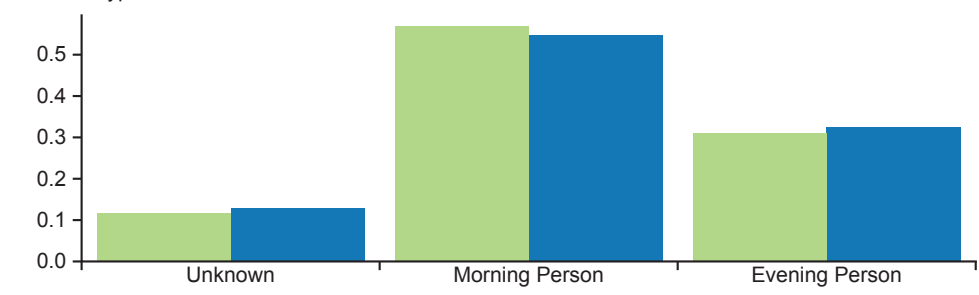

**D. Seasonality**

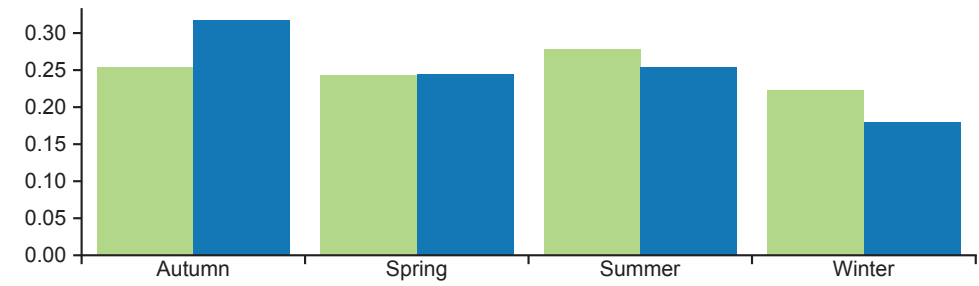

**E. Occupation**

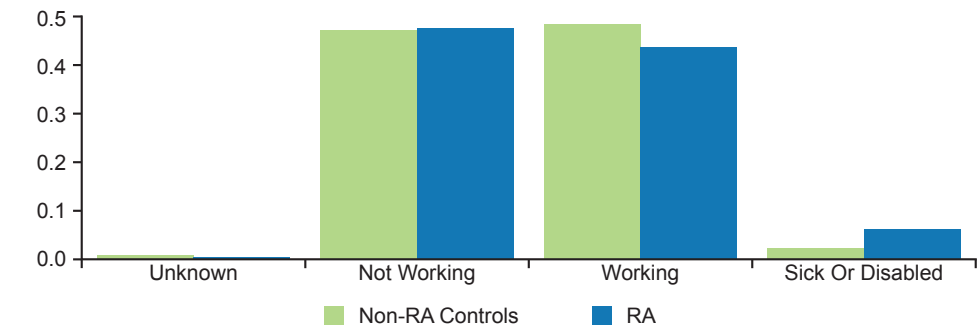

Supplement: S2 Fig — (PDF) [file pone.0319908.s002.pdf]

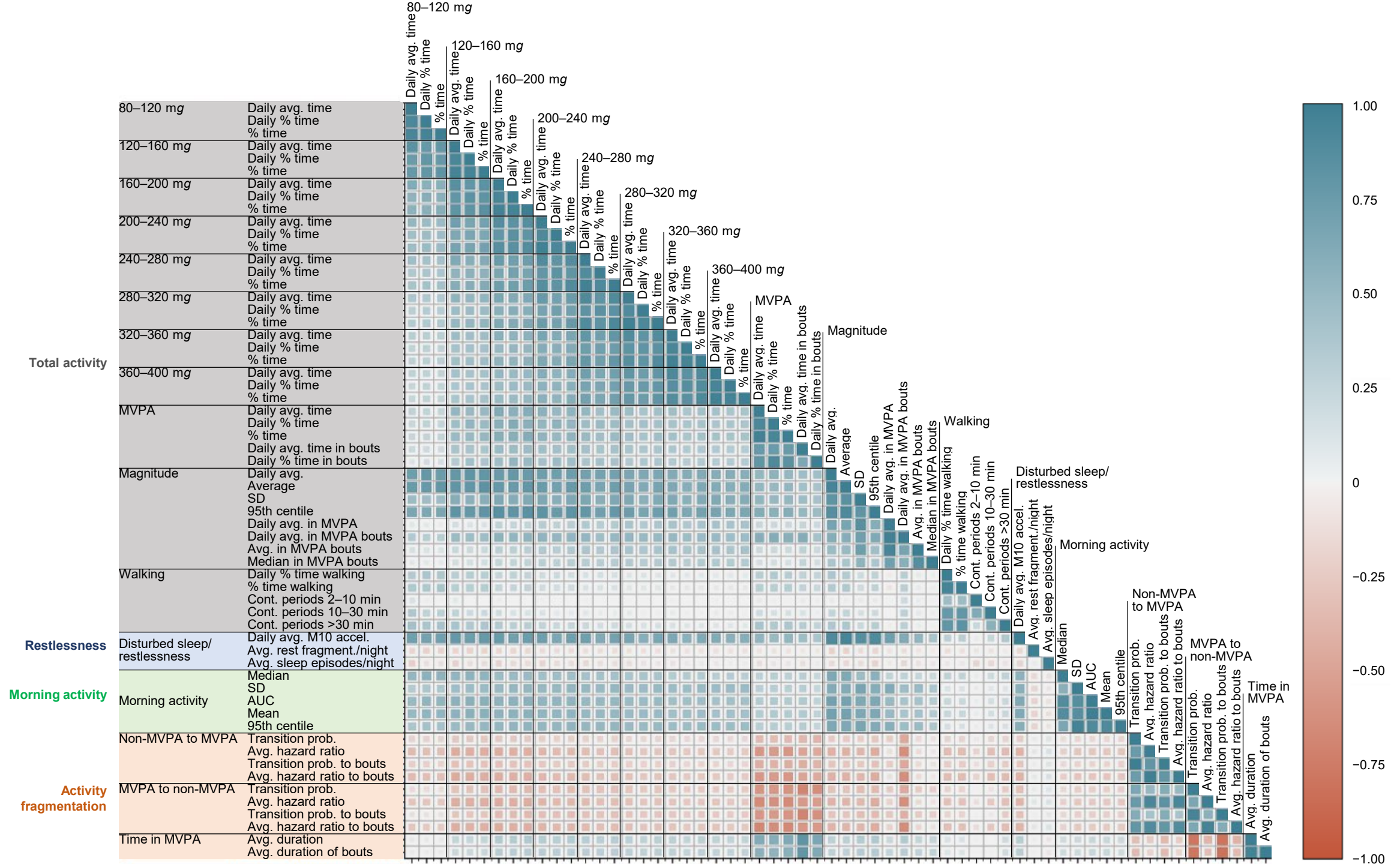

Supplement: S4 Fig — (PDF) [file pone.0319908.s004.pdf]

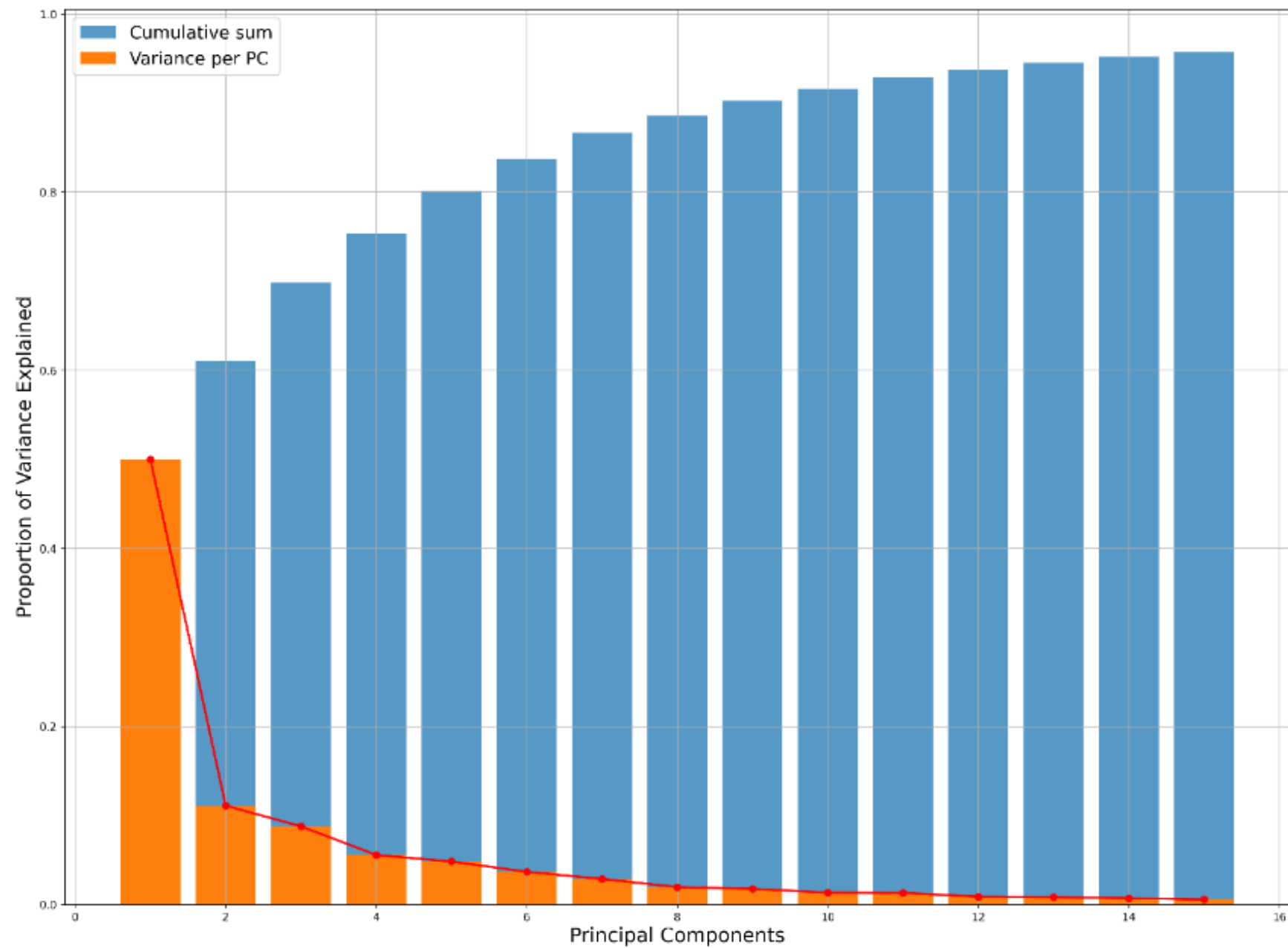

Supplement: S5 Fig — (PDF) [file pone.0319908.s005.pdf]
