# Supplementary material for: Assessment of physical activity patterns in patients with rheumatoid arthritis using the UK Biobank
Source: PLoS One. 2025 Mar 26;20(3):e0319908. doi: 10.1371/journal.pone.0319908 (PMC11940758; doi:10.1371/journal.pone.0319908)
Supplement: S3 Fig — (PDF) [file pone.0319908.s003.pdf]

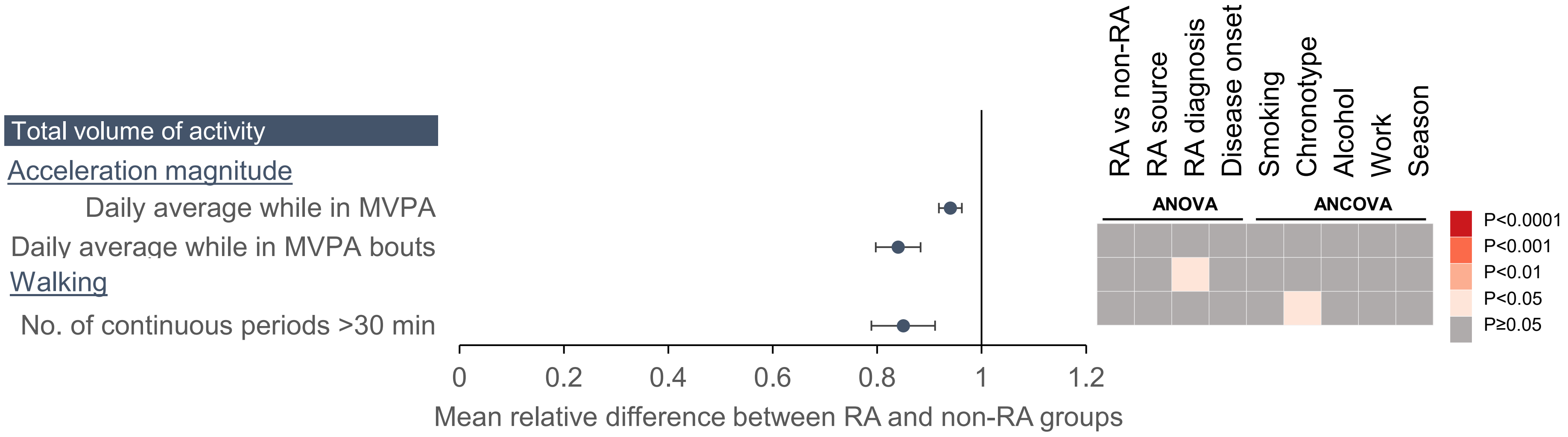
Total volume of activity
Acceleration magnitude
Daily average while in MVPA
Daily average while in MVPA bouts
Walking
No. of continuous periods >30 min
0
0.2
0.4
0.6
0.8
1
1.2
Mean relative difference between RA and non-RA groups
RA vs non-RA
RA source
RA diagnosis
Disease onset
Smoking
Chronotype
Alcohol
Work
Season
ANOVA
ANCOVA
P<0.0001
P<0.001
P<0.01
P<0.05
P≥0.05
