# Supplementary material for: Assessment of physical activity patterns in patients with rheumatoid arthritis using the UK Biobank
Source: PLoS One. 2025 Mar 26;20(3):e0319908. doi: 10.1371/journal.pone.0319908 (PMC11940758; doi:10.1371/journal.pone.0319908)
Supplement: S1 Table — (DOCX) [file pone.0319908.s006.docx]

S1 Table

| **Domain** | **Pre-processed summary data type** | **Metric** | **Shortlisted** |
| --- | --- | --- | --- |
| Total volume of activity | Acceleration magnitude | Average acceleration magnitude | Yes |
|  |  | Median acceleration magnitude | No |
|  |  | 95th centile of acceleration magnitude | Yes |
|  |  | SD of acceleration magnitude | Yes |
|  |  | Daily average acceleration magnitude | Yes |
|  | Intensity levels | Daily average total time sedentary | No |
|  |  | Daily average total time in light activity | No |
|  |  | Daily average total time in MVPA | Yes |
|  |  | Daily average total time in each 40 m*g* range | Yes |
|  |  | Percent of time sedentary | No |
|  |  | Percent of time in light activity | No |
|  |  | Percent of time in MVPA | Yes |
|  |  | Percent of time in each 40 m*g* range | No |
|  |  | Daily average percentage time sedentary | No |
|  |  | Daily average percentage time in light activity | No |
|  |  | Daily average percentage time in MVPA | Yes |
|  |  | Daily average percentage time in each 40 m*g* range | Yes |
|  |  | Daily average acceleration magnitude while in light activity | No |
|  |  | Daily average acceleration magnitude while in MVPA | No |
|  |  | Daily average acceleration magnitude while in each 40 m*g* range | Yes |
|  |  |  |  |
|  |  |  |  |
|  |  |  |  |
| Total volume of activity | Bouts | Daily average total time in active bouts | No |
|  |  | Daily average total time in sedentary bouts | No |
|  |  | Daily average total time in MVPA bouts | Yes |
|  |  | Percentage time in active bouts | No |
|  |  | Percentage time in MVPA bouts | No |
|  |  | Daily average percentage time in sedentary bouts | No |
|  |  | Daily average percentage time in active bouts | No |
|  |  | Daily average percentage time in MVPA bouts | Yes |
|  |  | Daily average acceleration magnitude while in active bouts | No |
|  |  | Average acceleration magnitude while in active bouts | No |
|  |  | Median acceleration magnitude while in active bouts | No |
|  |  | Daily average acceleration magnitude while in MVPA bouts | No |
|  |  | Average acceleration magnitude while in MVPA bouts | Yes |
|  |  | Median acceleration magnitude while in MVPA bouts | Yes |
|  | Activity classification | Percent time walking | Yes |
|  |  | Daily average percentage time walking | Yes |
|  |  | Number of cont. walking periods with duration 2–10 minutes (with up to 30-second rest period) | Yes |
|  |  | Number of cont. walking periods with duration 10–30 minutes (with up to 1-minute rest period) | Yes |
|  |  | Number of cont. walking periods with duration >30 minutes (with up to 1-minute rest period) | No |
| Morning activity; (symptom specific:  morning stiffness) | Acceleration magnitude | Daily average of acceleration magnitude during the first hour after getting up | No |
|  |  | Daily average of acceleration magnitude during the first 2 hours after getting up | Yes |
|  |  | Daily average of acceleration magnitude during the first 4 hours after getting up | No |
|  |  | Daily average of 95th centile of acceleration magnitude during the first hour after getting up | No |
|  |  | Daily average of 95th centile of acceleration magnitude during the first 2 hours after getting up | Yes |
|  |  | Daily average of 95th centile of acceleration magnitude during the first 4 hours after getting up | No |
|  |  | Daily average of SD of vector magnitude during the first hour after getting up | No |
|  |  | Daily average of SD of vector magnitude during the first 2 hours after getting up | Yes |
|  |  | Daily average of SD of vector magnitude during the first 4 hours after waking up | No |
| Night-time activity (symptom specific: disturbed sleep and  restlessness) | Activity classification | Daily average L5 time – midpoint time of the least active 5 hours | No |
|  |  | Daily average M10 time – midpoint time of the most-active 10 hours | Yes |
|  |  | Daily average MSleep time – midpoint time of night-time sleep window | No |
|  |  | Average night-time sleep windows duration | No |
|  |  | SD of night-time sleep windows duration | No |
|  |  | Average number of movement episodes per night-time sleep window | No |
|  |  | Average number of movement episodes per hour of night-time sleep windows | No |
|  |  | Average number of sleep episodes per night-time sleep window | Yes |
|  |  | Average percent night-time rest efficiency per night-time sleep window | No |
|  |  | Average night-time rest fragmentation per night-time sleep window | Yes |
|  |  | Average percent time of sleep per night-time sleep window | No |
|  |  | Average daily total duration of diurnal sleep periods | No |
| Activity fragmentation | Intensity levels | Average length of consecutive time active | No |
|  |  | Average length of consecutive time sedentary | No |
|  |  | Transition probability of active to sedentary | No |
|  |  | Transition probability of sedentary to active | No |
|  |  | Average hazard of active to sedentary | No |
|  |  | Average hazard of sedentary to active | No |
|  |  | Average length of consecutive time in MVPA | Yes |
|  |  | Transition probability of MVPA to non-MVPA | Yes |
|  |  | Transition probability of non-MVPA to MVPA | Yes |
|  |  | Average hazard MVPA to non-MVPA | Yes |
|  |  | Average hazard non-MVPA to MVPA | Yes |
|  | Bouts | Average length of active bouts | No |
|  |  | Average length of sedentary bouts | No |
|  |  | Transition probability of active to sedentary bouts | No |
|  |  | Transition probability of sedentary to active bouts | No |
|  |  | Average hazard of active to sedentary bouts | No |
|  |  |  |  |
|  |  |  |  |
| Activity fragmentation | Bouts | Average hazard of sedentary to active bouts | No |
|  |  | Average length of MVPA bouts | Yes |
|  |  | Transition probability of MVPA to non-MVPA bouts | Yes |
|  |  | Transition probability of non-MVPA to MVPA bouts | Yes |
|  |  | Average hazard MVPA to non-MVPA bouts | Yes |
|  |  | Average hazard non-MVPA to MVPA bouts | Yes |
|  |  | Ratio of time in active bouts to overall time active | No |
